# Biodiversity of Archaea and floral of two inland saltern ecosystems in the Alto Vinalopó Valley, Spain

**DOI:** 10.1186/1746-1448-6-10

**Published:** 2010-10-13

**Authors:** Basilio Zafrilla, Rosa M Martínez-Espinosa, María A Alonso, María J Bonete

**Affiliations:** 1 División de Bioquímica y Biología Molecular, Facultad de Ciencias, Universidad de Alicante, Ap. 99, E-03080 Alicante, Spain; 2 Departamento de Ciencias Ambientales y Recursos Naturales, Facultad de Ciencias, Universidad de Alicante, Ap. 99, E-03080 Alicante, Spain

## Abstract

**Background:**

The extraction of salt from seawater by means of coastal solar salterns is a very well-described process. Moreover, the characterization of these environments from ecological, biochemical and microbiological perspectives has become a key focus for many research groups all over the world over the last 20 years. In countries such as Spain, there are several examples of coastal solar salterns (mainly on the Mediterranean coast) and inland solar salterns, from which sodium chloride is obtained for human consumption. However, studies focused on the characterization of inland solar salterns are scarce and both the archaeal diversity and the plant communities inhabiting these environments remain poorly described.

**Results:**

Two of the inland solar salterns (termed Redonda and Penalva), located in the Alto Vinalopó Valley (Alicante, Spain), were characterized regarding their geological and physico-chemical characteristics and their archaeal and botanical biodiversity. A preliminary eukaryotic diversity survey was also performed using saline water. The chemical characterization of the brine has revealed that the salted groundwater extracted to fill these inland solar salterns is thalassohaline. The plant communities living in this environment are dominated by *Sarcocornia fruticosa *(L.) A.J. Scott, *Arthrocnemum macrostachyum *(Moris) K. Koch, *Suaeda vera *Forsk. ex Gmelin (*Amaranthaceae*) and several species of *Limonium *(Mill) and *Tamarix *(L). Archaeal diversity was analyzed and compared by polymerase chain reaction (PCR)-based molecular phylogenetic techniques. Most of the sequences recovered from environmental DNA samples are affiliated with haloarchaeal genera such as *Haloarcula, Halorubrum, Haloquadratum *and *Halobacterium*, and with an unclassified member of the Halobacteriaceae. The eukaryote *Dunaliella *was also present in the samples.

**Conclusions:**

To our knowledge, this study constitutes the first analysis centered on inland solar salterns located in the southeastern region of Spain. The results obtained revealed that the salt deposits of this region have marine origins. Plant communities typical of salt marshes are present in this ecosystem and members of the *Halobacteriaceae *family can be easily detected in the microbial populations of these habitats. Possible origins of the haloarchaea detected in this study are discussed.

## Background

Salts in general, and particularly sodium chloride, have played an important role in human history, not only because of their nutritional value but also because of their applications in several industrial processes and their impact on agricultural practices. Hypersaline lakes, salt marshes, hot springs, saline ponds and salt deposits are distributed throughout the world and constitute natural resources from which salt has been extracted for many centuries. For geologists, some of these environments represent a model system to study salt precipitation and related biogeochemical processes in shallow evaporitic environments [1].

In warm and arid areas, salt can be recovered from seawater thanks to several procedures that involve solar salterns. The solar salterns are filled with seawater that is concentrated gradually by the effects of the wind and temperature. Thus, solar salterns are restricted to areas such as Mediterranean regions, where the climate is characterized by periods during which evaporation exceeds precipitation. This process is especially rapid in summertime when highly salted water flows through an increasingly concentrated pool until ponds crystallize.

In addition to a high salt concentration, the environment of solar salterns is also characterized by its pH (range from 6 up to 11). Although halophilic microorganisms living in these environments are distributed among all three domains of life, it has been extensively reported that members of the *Halobacteriaceae *family constitute the dominant microbial population, especially in those environments where the NaCl concentration ranges from 20% (w/v) up to halite saturation (< 32% (w/v)) [2-5]. In fact, the red color associated with hypersaline lakes and ponds is mainly due to the pigmentation of halophilic archaea and the eukaryote *Dunaliella *[6]. However, this does not mean that these are the only inhabitants contributing to the red color of these environments. Populations of halophilic bacteria and other members of Eukarya are also present in saltern ponds [7,8]. To thrive under these conditions, halophilic archaea accumulate potassium ions inside their cells to balance the high salt content of the environment. This ability differentiates them from halophilic bacteria that usually accumulate compatible solutes (betaine, ectoine) to counteract the high external salt concentrations [9-11]. Nevertheless, it has been found that some halophilic bacteria such as *Salinibacter *use the same strategy as haloarchaea to cope with osmotic stress [7]. Because halophilic archaea are the predominant microorganisms in hot and hypersaline environments, it is possible that they sustain key metabolic cycles under these conditions; indeed the understanding of life under such extreme circumstances has become a key area of research recently [12,13].

By amplifying 16S rDNA sequences directly from environmental samples, halophilic archaea have been detected in different hypersaline environments such as the crystallizer ponds of a marine saltern [10,14], alkaline soda lakes [15] or sediments of hypersaline Antarctic lakes and coastal salt marshes [16,17]. Microbial research in saline environments is important for several reasons: i) there are potential biotechnological applications of halophilic microorganisms [18,19]; ii) knowledge about microbial diversity in terrestrial saline environments may shed light on the properties of salt deposits and saline environments found on Mars [20,21]; and iii) primitive life on earth might have started in this kind of extreme environment, so these systems are ideal to understand the evolution of the biosphere on Earth [22].

Regarding the biodiversity of these halophilic environments, it is also very interesting to analyze the nature of the plant communities because of the various mechanisms that they have developed to cope with such extreme saline conditions. Plant growth is highly limited by salty soil conditions. The soils surrounding solar salterns are characterized by a high salt concentration. This places limitations on plant growth because the soil's osmotic potential is low, making it difficult for the plants to extract water from the soil (hydric stress situations) and in order to get water throughthe roots, the plant's internal osmotic potential must be lower than that of the soil. Consequently, plants have developed mechanisms to adapt to high salt levels in soil.

The exploitation of saltworks has become a very successful industry, with large scale production in southeastern Spain where Solvay developed a method to produce caustic soda from NaCl in 1817 [23]. Salt obtained from plants has been used since the second half of the nineteenth century for the production of glass, dyes and soap. Although these plants are no longer used in industry today, they still have a great environmental value. Some irrigation methods, along with increased desertification in arid and semiarid regions, resulting in increases soil salinization. In response to these phenomena, some scientists have studied halophilic plants as a tool to manage soils that have undergone the salinization processes [24-29].

The United Nations' environmental program focuses on desertification processes and has drawn attention to the use of halophilic vegetation to restore degraded lands, provide food for livestock and fix some atmospheric carbon dioxide. In most cases, the aim is not to remove the salt from the ground so much as it is to allow for some tracts of land to be useful again. Planting halophilic species could be very useful in areas where salinization has been caused by human activities (thus making it impossible to sustain traditional agricultural activities). The most interesting aspect of these halophilic plants is that they can be irrigated even with sea water. In any case, a good vegetation cover protects the underlying soil against erosion, improving its structure and assisting drainage processes against capillary ascent [24-29].

In Alicante County (Spanish Mediterranean coast) there are many examples of well-characterized coastal solar salterns from which salt is extracted for human consumption [30]. However, the inland solar salterns located in this county remain poorly described to this day [31]. In the inland solar salterns, the groundwater interacts with underground salt deposits, resulting in an increase in water salinity. The saline water is then pumped into the ponds, where salt is extracted in a manner similar to that used in coastal solar salterns.

**Figure 1:**
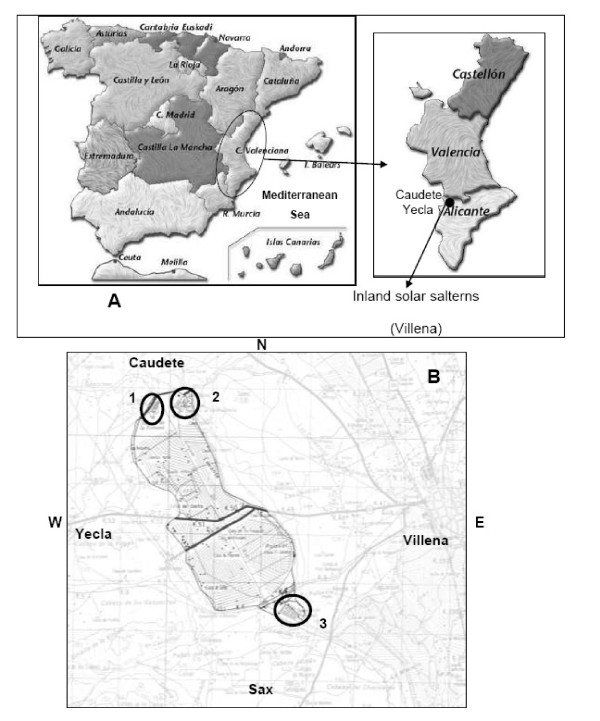
**Geographical location of the Alto Vinalopó Valley, Alicante, Spain (A)**. The three salterns are located at the margins of the old lagoon, called "La Laguna": 1, Fortuna; 2, Redonda; and 3, Penalva (B). Scale in panel B is 1:25,000.

The aim of this study was to describe general environmental aspects of two of the three inland solar salterns located in the municipality of Villena (northwest Alicante County, Figure 1). We have paid special attention to the ecological parameters and the properties of the collected brine; we have also assessed the Archaeal diversity of the crystallization ponds using (PCR)-based molecular phylogenetic approaches. Properties of the plant communities surrounding these inland solar salterns have also been studied in detail. Recently, this area has been catalogued as a "Wetland of interest" in the Alicante province http://www.cth.gva.es/areas/espacios/zonas_humedas/zon/Ficha-35.PDF, as it constitutes an optimum environment for a number of birds and salt marsh plant communities.

## Results and Discussion

### Description of the region

The valley where the solar salterns are located has an average altitude of 500 meters above sea level, with an approximate surface area of 94 Km^2^. Geomorphologically, this valley could be considered a corridor from the coast to the central regions of the Iberian Peninsula. Both sides of the valley are delimited by mountain ranges, but it is not crossed by any of these ranges. This results in a corridor valley phenomenon that increases the speed of winds running across the valley, which in turn also increases the evaporation rate in the "artificial shallow ponds". This is an important phenomenon for the salt extraction process, considering that, in the study region, evaporation is the most important contributing factor in the process of salt crystallization.

The valley studied in this work is the coldest, driest, and also the farthest inland in the Eastern Iberian Peninsula. The temperature ranges between -8 and 10°C in winter, and easily reaches 40°C and above in summer. These yearly temperature fluctuations are mainly due to the convergence of two meteorological phenomena in this zone. The first is known as "thermal inversion" and refers to overnight decreases in temperature in atmospheric layers that are in contact with the ground, resulting in dense fog banks that do not disappear until solar radiation heats the bottom layers or the winds are strong enough to disperse the fog [32,33]. The second meteorological phenomenon, known as "rain shadow", is related to the impact of mountain topography on rainfall. The valley is surrounded by mountains, which are low-pressure points that attract cloudy fronts and decrease recorded rainfall to less than 350 mm per year in the valley. Most of that scarce rainfall occurs in autumn and spring [31].

**Figure 2:**
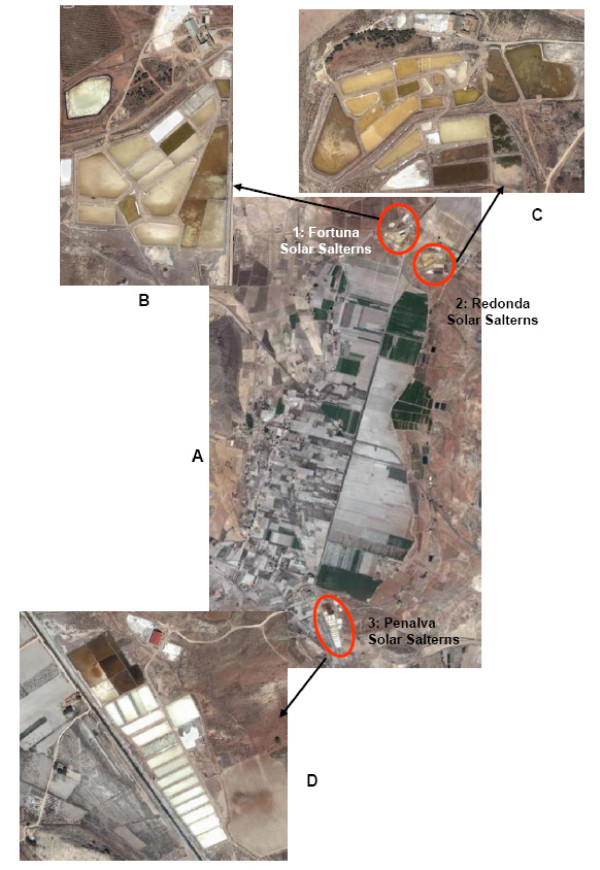
**A) Aerial view of the old lagoon, showing the locations of the saltern ponds (B, C and D)**.

The main geological formations observed in the geographical area of the Alto Vinalopó Valley are calcareous and dolomitic mountain ranges from the Cretaceous and Jurassic periods, whose sedimentation creates a quaternary valley. The valley is crossed by an alignment of Triassic diapires (Keuper facies) composed of red clay and gypsum, which constitute the geological source of the salinity. The diapiric phenomena originated between the Tertiary and Quaternary periods and formed a closed basin. Subterranean wells spilled water within these endorheic basins, forming the old lagoon known as "La Laguna" (Figures 1 &2), which has a surface area of around 15 km^2^. It was desiccated in 1803, but nowadays it is possible to distinguish the waterline because of the presence of fine gray salty slime. Most of this area is currently used for agriculture.

### Description of the Redonda and Penalva solar salterns

The salterns of Redonda, Fortuna and Penalva are located quite close to the perimeter of the aforementioned old lagoon. They are composed of several artificial shallow ponds and have been used for the last few centuries to obtain sodium chloride for human consumption. The present study focuses mainly on the Redonda and Penalva salterns (Figure 1B, Figure 2). These constitute around 1.25% of the overall area of solar salterns in the Alicante region, and along with the inland solar salterns located in the municipality of Salinas, they are the only inland evaporation systems in this region.

Currently, only the Penalva ponds are being used for NaCl production. The Redonda and Fortuna crystallizing ponds are the oldest in this region, which explains their irregular shapes (Figure 2).

In the Penalva salterns, a distinction can be made between heating and crystallizing ponds. The heating ponds are filled with salty water pumped from ground soil. As previously mentioned, saline water results from the interaction between groundwater and salt deposits, and the salt concentration at the pumping point, as estimated by a Baumé hydrometer, is 10°Bé which corresponds to 10% salt. In summertime (from May to September) especially, the water evaporation process becomes very rapid, resulting in an increased salt concentration. The water then turns into brine, which is driven from heating ponds to crystallizing ponds where NaCl precipitates when the total salt concentration increases to just above 300 g/l.

### Physico-chemical characterization of the saline water and brine from the Redonda and Penalva salterns

To shed light on the characterization of these inland solar salterns, we used different approaches, such as physico-chemical characterization of the saline water and brine samples. Several parameters, such as temperature, pH and conductivity, were measured in the field immediately before the collection of water samples from the Redonda and Penalva ponds. The results summarized in table 1 show that, in both cases, the pH is quite close to neutrality and there is no spatial or temporal variation in pH. Samples obtained from the center of the ponds also exhibited the same conductivity and salinity as those obtained from the margins; however, the optical density values of the margin samples were higher due to the accumulation of *Dunaliella *caused by the winds, as confirmed by optical microscopy analysis. Conductivity and salinity values obtained from the Redonda ponds were similar to those obtained from Penalva. Conductivity, or specific conductance, is a measure of the ability of the solution to carry an electrical current; in general, the greater the salinity, the greater the conductivity. However, this salinity-conductivity relationship is not straightforward because the same conductivity values can be obtained from samples with different ion compositions and concentrations [34]. This is because conductivity is a function of the specific ions present in the solution, as well as the level of concentration of the ions. For this reason, complete chemical analyses of saline water and brine were undertaken.

**Table 1:** Physico-Chemical characterization of the samples.

Parameters	Redonda ponds	Penalva ponds
**Temperature **(°C)	30	32

**pH**	7.5	7.5

**Conductivity** (mS cm^-1 ^at 25°C)	226 ± 15	221 ± 24

**Salinity **(ppt)	145 ± 10	142 ± 15

**O.D**. (600 nm)	0.000* 1.937**	0.000* 1.235**

Average values for several parameters obtained from salted water collected from the surface of the crystallizer ponds at the Redonda and Penalva inland salterns; parameters were measured at the time of sample collection. Water from the center (*) and margins (**) of the ponds.

Chemical analysis of the saline water samples from Penalva revealed that the main cations were Na^+ ^(121 g/l), Ca^2+ ^(0.6 g/l) and Mg^2+ ^(4 g/l); while Cl^- ^(187 g/l) and SO_4_^2- ^(21 g/l) were the main anions detected. Other trace elements such as potassium and manganese were also found. It is also important to note that non-negligible concentrations of CaSO_4 _(2 g/l), MgSO_4 _(22 g/l) and MgCl_2 _(0.1 g/l) were detected. Similar results were obtained from the Redonda salterns. Hypersaline waters are defined as those with total salt concentrations greater than that of seawater (i.e., >3.5%). On the basis of the chemical composition of the water, saline water can be classified into two categories: *Thalassohaline *water (derived from seawater, with Na^+ ^and Cl^- ^as the predominant ions) and *Athalassohaline *(with an ionic composition markedly influenced by the area where the pond develops) [35]. Athalassohaline waters usually contain higher concentrations of bivalent ions such as calcium, magnesium and sulfate, in contrast to the relative dominance of monovalent ions (sodium and chloride) in seawater [22,35]. The results obtained from chemical analysis of saline water samples from the Redonda and Fortuna salterns, which clearly show that Na^+ ^and Cl^- ^are the dominant ions, indicate that these are thalassohaline environments.

Brine samples from the Penalva salterns, which had a pH value of around 7.8, were collected to estimate their inorganic composition, as described in the Methods section. The results obtained showed that 99.8% of the brine salt is NaCl, which has been collected for human consumption. The hypersaline brine obtained had a final composition similar to that reported for brine obtained in coastal solar salterns [35], where calcite (CaCO_3_), gypsum (CaSO_4_.2H_2_O), halite (NaCl), sylvite (KCl) and carnallite (KCl.MgCl_2_.6H_2_O) precipitate out sequentially as evaporation occurs. Therefore, the data obtained from these chemical analyses strongly support a seawater origin for the salt deposits of the Alto Vinalopó Valley.

### Analysis of the plant communities

In salt marshes all around the world, halophytic plant communities usually are present low species diversity. This is due to the saline soils, whose osmotic effect inhibits mesophilic plant growth by reducing the ability of the plants to take up groundwater. The effects of excess ion concentration on plant cells have also been very well described [[36]; see also Table 1 in reference [35]].

The studied area has a very dry climate, which is a determining factor in the landscape of the areas around the Penalva and Redonda Salt marshes. In these areas, the landscape shows low-density and high-diversity communities, unlike the salt marshes, where plant communities are dense and present low diversity with a high production rate.

A total number of 20 halophylous taxa were identified. These taxa are members of eight different botanical families. *Plumbaginaceae *and *Amaranthaceae *are highly represented, with seven and five species, respectively (Table 2).

**Table 2:** List of plants found in the studied marshes.

Species	Family
*Arthrocnemum macrostachyum*	*Amaranthaceae*

*Sarcocornia fruticosa*	*Amaranthaceae*

*Atriplex glauca*	*Amaranthaceae*

*Halimione portulacoides*	*Amaranthaceae*

*Suaeda vera*	*Amaranthaceae*

*Limonium caesium*	*Plumbaginaceae*

*Limonium cossonianum*	*Plumbaginaceae*

*Limonium delicatulum*	*Plumbaginaceae*

*Limonium parvibracteatum*	*Plumbaginaceae*

*Limonium supinum*	*Plumbaginaceae*

*Limonium thiniense*	*Plumbaginaceae*

*Limonioum xeugeniae*	*Plumbaginaceae*

*Tamarix canariensis*	*Tamaricaceae*

*Tamarix boveana*	*Tamaricaceae*

*Puccinellia fasciculata*	*Poaceae*

*Cynomorium coccineum*	*Cynomoriaceae*

*Frankenia corymbosa*	*Frankeniaceae*

*Inula crithmoides*	*Asteraceae*

*Juncus maritimus*	*Juncaceae*

In examining the adaptation strategies of halophytes, we can distinguish two principal approaches.

**Figure 3:**
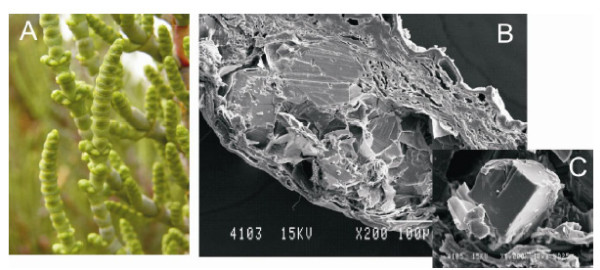
**Succulency of *Sarcocornia fruticosa *(A)**. SEM of transverse section of *Sarcocornia *stem with sodium chloride in the parenchyma (B). Detail of sodium chloride (NaCl) (C). Bar scales are included in the pictures.

Some species are able to decrease their intracellular osmotic potential by storing salt or just by synthesizing soluble sugars such as sucrose or glucose which are also stored within the cells [37]. Without these special adaptations, halophytes would not be able to obtain the water that they need to survive. The species displaying such adaptations in the studied locations are: *Arthrocnemum macrostachyum*; *Sarcocornia fruticosa *(Figure 3) and *Suaeda vera*. As a result of this physiological adaptation, these species present a succulent morphology, which allows them to maintain control over the progressive accumulation of salt. Such succulent morphology is only found in plants that live in chloride-rich soils. The storage of chloride ions causes the cytoplasm to swell, giving plants their characteristic appearance (succulence). This succulence could be due in part to the nature of the proteins, which usually are halophilic and present a highly negative surface charge [38,39]. This property makes the proteins more soluble and flexible at high salt concentrations. Nevertheless, the high surface charge has to be neutralized, mainly by tightly-bound water dipoles that probably contribute to the accumulation of water. Halophilic plants living in soils in which chloride is not the main ion do not present succulent morphology. By contrast, these ionic environments exert the opposite effect on cytoplasmic proteins [40].

**Figure 4:**
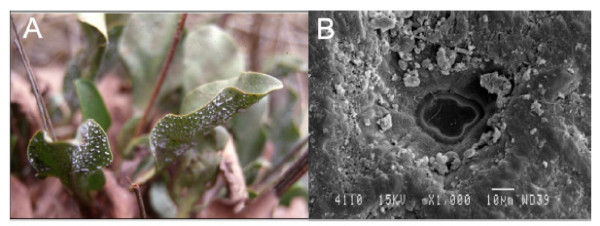
**Leaves of the genus *Limonium *with salt secretions (A)**. SEM of salt gland (B). Bar scale is included in panel B.

In the second type of adaptation, some species are able to secrete salt through leaf glands (e.g., the vesicle hairs on the *Atriplex *genus or other types of glands found in the *Limonium *(Figure 4), *Tamarix *or *Frankenia *genera).

Before examining the plant communities, it is necessary to consider that plants also adapt to periodic fluctuations in salt concentrations in the soil, and respond differently to different ions. These fluctuations are influenced by factors such as temperature, humidity and the salt concentration and composition. For these reasons, the plant communities observed in the Penalva and Redonda ecosystems are distributed according to the characteristics of the soil.

*Arthrocnemum macrostachyum *communities grow in drier soils with higher salt concentrations. When the land becomes wetter, *Sarcocornia fruticosa *communities develop. Both of these *Amaranthaceae *are succulent species with a high biomass production and represent a very important vegetation cover.

**Figure 5:**
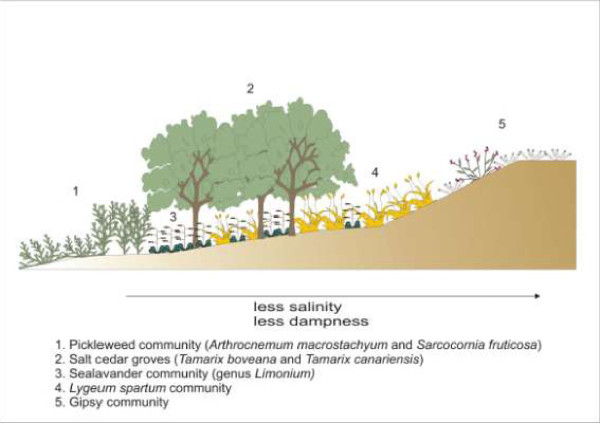
**Effects of soil salinity on the spatial distribution of halophytic vegetation**.

There are some other non-succulent halophilic plants that can grow in the salty soils surrounding the Penalva and Redonda ponds. These plants do not produce a high biomass but they play an important role in biodiversity in this kind of ecosystem. The most important genera are *Limonium*, *Tamarix *and *Atriplex*, all of which present a very high degree of speciation. In both ecosystems (Penalva and Redonda), it is possible to observe seven different *Limonium *species. (*L. supinum, L. thiniense, L. delicatulum, L. cossonianum, L. parvibracteatum, L. caesium *and *Limonium xeugeniae*). All seven *Limonium *species are restricted to the Ibero-Levantine area and are included in the Plant Red List [41]. It is important to note that other endemic halophilic plants such as *Atriplex glauca *or *Frankenia corymbosa *are also present. It is even possible to observe the halophilic parasite *Cynomorium coccineum *growing close to *Suaeda vera, Atriplex glauca *or *Halimione portulacoides*. The most developed plant communities in these analyzed habitats are small forests dominated by *Tamarix *species (*T. boveana, y T. canariensis*) (Figure 5).

### Analysis of Archaeal diversity

In view of the archaeal-specific primers used and the repeated isolation of haloarchaea from similar environments, we expected our amplification products to be haloarchaeal. Haloarchaeal sequences were indeed identified from both inland saltern systems (Redonda and Penalva). The BLAST comparisons revealed the existence of several genera belonging to the *Halobaceriaceae *family, which has colonized these artificial environments.

**Figure 6:**
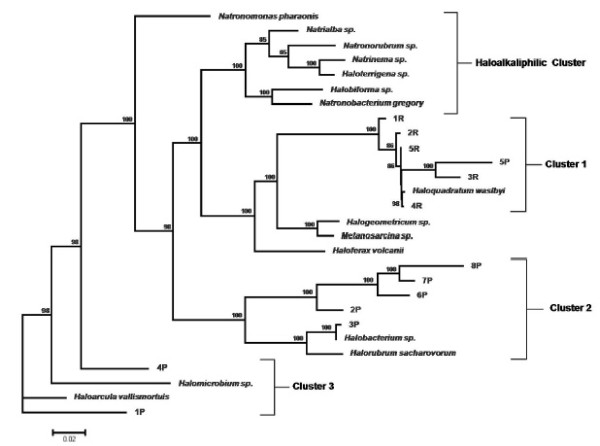
**Phylogenetic tree inferred from 16S rRNA gene sequences**. Sequences from the library clones from the salty water have been included. Numbers at nodes are bootstrap confidence values expressed as percentage of 500 bootstrap replications. Bootstrap values higher than 80% are indicated in the main nodes. Bar, 0.02 expected nucleotide substitutions per site.

The phylogenetic tree shown in figure 6 represents phylotypes obtained in this study and their closest relatives. Most of the sequences obtained from the Redonda ponds (1R-5R) were grouped into cluster 1, which showed the highest degree of similarity to *Haloquadratum walsbyi *(98-99% similarity). However, the highest degree of diversity was found in the Penalva samples. In this case, the 16S rRNA gene sequences were similar to those of the genera *Haloarcula *(1P, 93% similarity), *Haloquadratum *(5P, 93% similarity) and *Halobacterium *(3P, 99% similarity), as well as an unclassified member of the *Halobacteriaceae *(represented by sequences 2P, 6P-8P; 96-98% similarity). Other haloarchaea such as *Haloferax *were not detected based on the 16S rRNA sequences. Studies conducted in an Australian crystallizer pond found that, although *Haloferax *can frequently be isolated on plates, it was not detected based on 16S rRNA sequences, mainly because it is not a dominant group [42]. These results suggest that haloarchaea as well as several halophilic bacteria are the dominant microbial flora in hypersaline waters with near-saturation salt levels [42]. Although this study is does not focus on halophilic bacterial communities, research on this topic should be conducted in inland solar salterns.

In summary, six of the thirteen sequences were identified as *Haloquadratum walsbyi*. This square-shaped microbe constitutes the dominant fraction of the microbial community in the crystallizers of Santa Pola (on the Mediterranean coast, Alicante region) [43], supporting the idea that this organism constitutes the dominant population in hypersaline environments. Nevertheless, the archaeal communities inhabiting these environments could be very diverse on the basis of the nutrient-enriched source water or weather conditions. For example, *H. walsbyi*, which has been detected in this study, represents only a minor fraction of the sequences recovered from Adriatic solar salterns [44].

Considering that these ponds are artificial systems, it is quite interesting to look for the origin of the archaeal populations. Several research groups all over the world have isolated microorganisms from rock salt or halite brine that suggest that microorganisms, mainly halophiles, may survive over geological time in evaporite deposits, although they are not necessarily as old as the formations themselves [45,46]. As haloarchaea remain viable within halite crystals for considerable periods of time [35], it is possible that haloarchaea detected in Redonda and Penalva could have been entrapped within fluid inclusions in halite deposits. This possibility has been previously suggested by other authors, who have demonstrated that haloarchaea can remain "alive" within such fluid inclusions rather than within the halite crystal structure [47].

Eukaryotes in general are scarce in environments with greater than 20% (w/v) salt concentrations, with the exception of phototrophic flagellates of the genus *Dunaliella *or the brine shrimp *Artemia salina*, which frequently impart pigmentation to brine [48]. *Dunaliella *cells were harvested from Penalva and Redonda saline water, as described in the Methods section. High concentrations of these organisms were collected in samples from the upwind side of the ponds. The red color that the ponds display mainly in summer is due to carotenoid pigments that protect cells from the harmful effects of ultraviolet light [49] or to the retinal protein bacteriorhodopsin. However, *Artemina salina *was not detected during the sampling season.

## Conclusions

This work contributes to our knowledge of inland solar salterns located in the south-eastern region of Spain. The biodiversity and location of plants were clearly determined by the soil's salt concentration and composition. The adaptations described here allow plant communities to inhabit salterns, raising the possibility of their use in soil bioremediation, which could be of great interest in regions where desertification has become a major problem. The ponds, characterized by their red color in summer, provide perfect ecosystems for the growth of haloarchaea and *Dunaliella*. Along these lines, the micro-biodiversity described in the Redonda and Penalva ponds is similar to that described in coastal solar salterns. This is an expected result, considering that the saline water was found to be thalassohaline. A most interesting question is whether the halophilic microorganisms isolated from seaside or coastal solar ponds are the descendants of populations that became trapped in salt when the evaporites formed millions of years ago. The survival of haloarchaea or other halophilic microorganisms in a state of dormancy over geological time periods remains to be proven unequivocally, and the salt ponds described here could provide a good model to analyze this important question.

## Methods

### Location of the study region

The salterns of Redonda, Fortuna and Penalva are located in a border zone between the regions of Alicante, Albacete, and Murcia (northwest Alicante Province, Spain; Redonda salterns: 38°39'16.23''N, 00°55'45.77''W; Penalva salterns: 38°36'58.20''N, 00°54'16.12''W).

### Collection and physico-chemical analysis of the samples

Water from the center and the margins of six crystallizing ponds in each saltern (Redonda and Fortuna) was collected during July and September 2008; the water was collected in sterile 15-ml flasks and kept aseptically at 4° until analysis. Samples were stored for no longer than 48 hours before analysis. Brine from the Penalva ponds was collected between of August and September 2008. The temperature and pH of the saline water were measured in the field by means of a portable conductimeter (Mettler Toledo, Mod Seven Easy) and portable pH meter (Mettler Toledo, Mod Seven Easy). Measures of the specific gravity of the water were also undertaken in the field, using a Baumé hydrometer. From these measurements, the salt concentration of the samples was estimated based on correlations between Baumé degrees and NaCl concentration for different solutions, taking into account that a hydrometer placed in pure water reads 0°Bé (0% salts). All of the parameters were measured three times to get average values and errors. To analyze the optical density of the samples, water was collected from both the center and the margins of the ponds, as the water in these areas differed in color.

For the chemical characterization procedures, 1 ml from each sample was used to determine the inorganic ion composition and concentration (Na^+^, Ca^2+^, Mg^2+^, Cl^-^, SO_4_^2-^), following UNE standard methods (Spanish Association for Standardization and Certification: http://www.aenor.es/desarrollo/normalizacion/normas/buscadornormas.asp?pag=p): UNE 34204:1981; UNE EN 77041:2002; UNE 34233:1984

### DNA isolation

DNA was isolated from environmental samples using the protocol summarized in the Halohandbook [50]: total DNA was isolated from six different samples from each saltern (Redonda and Penalva) by passing 5 ml of each sample through a nitrocellulose filter (0.2 μm, Ministar) and resuspending the organic material in 500 μl of ultrapure distilled water. After incubation at 100°C for 15 min, the samples were centrifuged (13,000 rpm for 5 min). The lysate supernatant was extracted with an equal volume of buffered phenol. This step was repeated twice. Finally, 100 μl of aqueous phase was used for the amplification of 16S rRNA.

### PCR amplification and construction of rDNA and clone libraries

Archaeal 16S rRNA genes were amplified using the GenAmp PCR System 2700 system (Applied Biosystems) with the following program: 95°C (5 min) followed by 35 cycles (1 min denaturation at 95°C, 1 min annealing at 54°C and 2 min elongation at 72°C, with a final extension step at 72°C for 8 min). The archaeal-specific primers were: 16SFor (5'-TTCCGGTTGATCCTGCGGGA-3') [51] and 16SRev (5'-GGTTACCTTGTTACGACT-3') [52]. Each reaction (50 μl) contained 200 nM of the primers, 0.2 mM dNTPs, 3 mM MgCl_2_, 4 μl of PCR buffer, 125 ng DNA and 2 U Taq polymerase. The amplification products were purified from a 1% agarose gel using a GFX kit from Healthcare. Then they were ligated into a pSTBlue-1 Acceptor Vector (pSTBlue-1 Acceptor Vector Kit, from Novagen) and subsequently used to transform *E. coli *NOVABLUE cells. The resulting clones were screened for 16S rDNA inserts by colony PCR using 20 pmol M13 forward and M13 reverse primers.

### 16S rRNA gene sequencing and phylogenetic analyses

Archaeal 16S rRNA genes were sequenced using M13For (5'-TGTAAAACGACGACGGCCAGT-3'), M13Rev (5'-ATGACCATGATTACGCC-3') and 16Sec (5'-TTATTGGGCCTAAAGCGTCCGTAG-3') primers. The amplified sequences were cloned into a pGEM vector (pGEM^®^-T Easy Vector kit, from PROMEGA) and sequenced using a Big Dye Sequencing kit ver. 3.1 (Applied Biosystems) and an ABI PRISM 3100 sequencer (Applied Biosystems). The EMBL accession numbers for the nucleotide sequences determined in this work are FN669141 (1P), FN669142 (2P), FN669143 (3P), FN669144 (4P), FN669145 (5P), FN669146 (6), FN669147 (7P), FN669148 (8P), FN669149 (1R), FN669150 (2R), FN669151 (3R), FN669152 (4R) and FN669153 (5R).

Relevant 16S rRNA sequences from halophilic haloarchaea were obtained from GenBank http://www.ncbi.nlm.nih.gov using BLASTN and BLASTP. Alignments of the 16S rRNA sequences were created using CLUSTALW.

Tree topologies were evaluated using the maximum likelihood (fastDNAml) method.

### Assessment of the presence of *Dunaliella *and *Artemia *in samples

A volume of 0.5 ml from each sample was used to assess the presence of *Dunaliella *and *Artemia *by optical microscopy with an Axiostar Zeiss microscope or a magnifying glass, respectively.

### Parenchymal images

For scanning electron microscopy (SEM), parenchymal samples from *Sarcocornia *and *Limonium *were coated with gold for 5-10 min and examined under a JEOL-840 microscope.

### Plants identification

To study the floral community, the traditional botanic system has been used: plants were collected and dried to store them at the herbarium (ABH) [53] located at the University of Alicante. To obtain proper taxa nomenclature, methods proposed by Tutin et al. [54], Castroviejo et al. [55], Bolòs et al. [56] and Mateo and Crespo [57] were followed.

## Competing interests

The authors declare that they have no competing interests.

## Authors' contributions

BZ did the fieldwork, the PCR amplification of rDNA genes, the library construction and the phylogenetic and chemical analyses of the samples. RMME compiled the historical, geological and ecological data related to the Fortuna and Penalva inland solar salterns, and co-wrote the manuscript. MAA was involved in the flora characterization as well as in the description of the habitat. MJB designed the study and co-wrote the manuscript. All authors have read and approved the final manuscript.
